# Active Fault Localization of Actuators on Torpedo-Shaped Autonomous Underwater Vehicles

**DOI:** 10.3390/s21020476

**Published:** 2021-01-11

**Authors:** Fuqiang Liu, Yan Long, Jun Luo, Huayan Pu, Chaoqun Duan, Songyi Zhong

**Affiliations:** 1 State Key Laboratory of Mechanical Transmission, College of Mechanical Engineering, Chongqing University, Chongqing 400044, China; longy@cqu.edu.cn (Y.L.); luojun@shu.edu.cn (J.L.); 2 School of Mechatronic Engineering and Automation, Shanghai University, Shanghai 200444, China; phygood_2001@shu.edu.cn (H.P.); chaoqun.duan@hotmail.com (C.D.); zhongsongyi@shu.edu.cn (S.Z.)

**Keywords:** autonomous underwater vehicle (AUV), actuator fault, fault diagnosis (FD), fault localization (FL), feature analysis

## Abstract

To ensure the mission implementation of Autonomous Underwater Vehicles (AUVs), faults occurring on actuators should be detected and located promptly; therefore, reliable control strategies and inputs can be effectively provided. In this paper, faults occurring on the propulsion and attitude control systems of a torpedo-shaped AUV are analyzed and located while fault features may induce confusions for conventional fault localization (FL). Selective features of defined fault parameters are assorted as necessary conditions against different faulty actuators and synthesized in a fault tree subsequently to state the sufficiency towards possible abnormal parts. By matching fault features with those of estimated fault parameters, suspected faulty sections are located. Thereafter, active FL strategies that analyze the related fault parameters after executing purposive actuator control are proposed to provide precise fault location. Moreover, the generality of the proposed methods is analyzed to support extensive implementations. Simulations based on finite element analysis against a torpedo-shaped AUV with actuator faults are carried out to illustrate the effectiveness of the proposed methods.

## 1. Introduction

Autonomous Underwater Vehicles (AUVs) belonging to unmanned marine robots are preferable for exploring complex underwater circumstances for mankind. Actuators, particularly referred to propeller and rudders on torpedo-shaped AUVs, are generally attached to provide propulsion and attitude control forces and torques [1]. Unanticipated factors inside and outside the AUV hull could cause countless disadvantageous effects, of which some may alter the desired forces and/or torques and result in mission failure [2].

In principle, fault diagnosis (FD) and fault-tolerant control (FTC) deployed on AUVs could improve their survivability and optimize the mission execution in response to faults. Real-time FD should be an early action to expose system faults [3], which probably provide information of fault severity and location for FTC to ensure the system stability [4,5]. Research efforts on AUV FD have been carried out for several decades facing the faults of actuators, sensors, and hardware/software, where the methods are typically assorted into categories of qualitative and quantitative analyses [6]. Various methods could be adopted to diagnose system faults with or without models qualitatively [7]. For instance, pattern recognition against actual and predicted sequences based on grey qualitative simulation was used to diagnose thruster faults [8]; a fault tree model and fuzzy neural network were adopted to support the risk analysis of various faults existed on AUV subsystems [9]; self-organizing maps and fuzzy logic clustering methods were used to isolate internal and external thruster faults through fault detector units [10]. Usually, quantified fault information and definite isolation of faulty components are not provided in qualitative analysis, but these are necessary for active FTC.

To provide accurate fault information for early and effective FTC, data-driven and model-based methods were developed where the relationships between inputs and outputs were quantitatively expressed [11]. Recent surveys of quantitative methods have been thoroughly reflected in [12]. The data-driven methods classify normal and faulty situations by identifying data patterns statistically, e.g., a novel data-driven algorithm was developed recently by integrating techniques of fast Fourier transform and uncorrelated multi-linear principal component analysis, which could achieve effective space visualization for FD under actuator and sensor faults [13]. Some other methods of implementations include recursive neural networks [14], online Bayesian nonparametric technique [15], wavelet-based filtering method [16], and energy-aware architecture [17]. Within these methods, the acquired data need to represent the fault types related to the investigated objects. Comparatively, the model-based methods generally approximate the system dynamics and parametrically describe the fault extents where various techniques support the identification of the discrepancies. For instance, a switching-mode hidden Markov model was used to describe the faulty system, and a particle filter was adopted to isolate the faults [18]; the Fossen model was used to describe the ODIN AUV, and a dedicated bank of scalar filters based on the nonlinear geometric approach was built to simultaneously detect and isolate the actuator faults [19]. Some other research findings adopted the sliding-mode approach [20], structural analysis [21], improved Gaussian particle filtering [22], adaptive-threshold observing [23], generalized-likelihood-ratio comparison [24], and Livingstone 2 diagnosis engine [25]. Performance of the model-based FD is restricted by the model accuracy, which relies on expert knowledge. Moreover, the obtained features would probably confuse the isolation processes since a fault symptom may correspond to more than one fault.

The aforementioned research provides various solutions for actuator FD. However, less attention has been paid to the issues of fault localization (FL) among the propeller and rudders of the torpedo-shaped AUVs as confusions of fault features could occur. In [26], the deformation fault on a rudder corner was isolated by qualitative analysis. However, the assumed deformation only covers a small number of faults that could occur on a torpedo-shaped AUV; moreover, the problem of feature confusion has not been involved. The methods in this paper locate the faults among actuators of the torpedo-shaped AUVs by absorbing the advantages of qualitative and quantitative methods. When any influential fault occurs, the dynamic inputs (forces and torques) acting on the AUV deviate from the required ones. To accurately locate the faulty component, possible faulty situations of different regions are analyzed, and necessary fault features are generated correspondingly. By synthesizing all the analyzed situations, feature sufficiency is promised; hence, a qualitative FL strategy using feature matching is proposed to reveal suspected faulty components. To further locate the faulty component under feature confusion, active FL strategies with purposeful actuator control are proposed, which can definitely isolate the fault [27]. Subsequently, the generality of the proposed methods is analyzed to face measurement noise and typical robots. Simulations by using ANSYS Fluent are developed to illustrate the effectiveness of the proposed methods.

The remainder of this paper is organized as follows. Section 2 formulates the problem. Section 3 presents the main results, including the fault feature extraction, FL proposition, and generality analysis. In Section 4, simulations of a torpedo-shaped AUV with propeller and rudder faults are presented. Section 5 concludes this paper.

## 2. Problem Formulation and Preliminaries

In this section, the dynamic model and the actuator configurations of a torpedo-shaped AUV will be presented first. Afterwards, the problem will be formulated.

### 2.1. Dynamic Model

**Figure 1:**
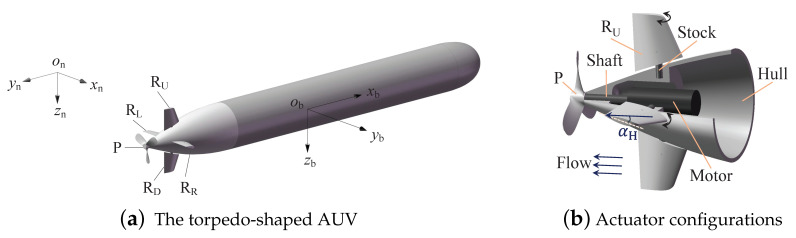
A torpedo-shaped AUV (**a**) and the actuators (**b**), where $\alpha_{H}$ is a rudder angle from the plane $x_{b}o_{b}y_{b}$.

The torpedo-shaped AUV is shown in Figure 1a, where a North-East-Down (NED) coordinate system {n} and a body-fixed reference frame {b} are displayed simultaneously.

Typically, a torpedo-shaped AUV is motivated by two kinds of actuators, a propeller and a rudder (Figure 1b), where the propeller (P) with several pieces of blades provides a thrust along axis $x_{b}$, and four pieces of rudder surfaces (the horizontal $R_{L}$ and $R_{R}$ and the vertical $R_{U}$ and $R_{D}$) that are installed near the AUV rear form a cross to control and stabilize the attitudes of yaw, pitch, and roll. To describe the AUV nonlinear dynamics, the following two equations are usually adopted [28]:

(1) $$\begin{array}{c} \left\{\begin{array}{c} M\dot{\nu }+C\left(\nu \right)\nu +D\left(\nu \right)\nu +g\left(\eta \right)=\tau ,\\ \dot{\eta }=J\left(\eta \right)\nu , \end{array}\right. \end{array}$$

where $\nu \in R^{6\times 1}$ includes the generalized velocities defined in {b}; $\eta \in R^{6\times 1}$ contains the generalized positions and heading angles defined in {n}; $\tau \in R^{6\times 1}$ is the dynamic control input containing 3 forces and 3 torques; *M*, C(ν), and D(ν) denote inertia, Coriolis, and damping matrices, respectively; g(η) contains generalized gravitational and buoyancy forces; J(η) is a coordinate revolution matrix between {n} and {b}.

The relationship between the actuator input u and the dynamic control input τ for a torpedo-shaped AUV is

(2) $$\begin{array}{c} \tau =Bu, \end{array}$$

where *B* is the configuration matrix. The positive or negative features of all rudder angles in u are defined based on whether the generated lift forces could produce correspondingly positive or negative attitude angles.

### 2.2. Problem Formulation

If an actuator gets faulty, some elements in *B* and u of (2) are changed first, and the influences are then transferred to τ, which introduces control losses for the AUV. The changes of τ, expressed as additive fault parameter $f_{\tau }≜{\left[f_{X},\;f_{Y},\;f_{Z},\;f_{K},\;f_{M},\;f_{N}\right]}^{T}$, can be estimated by the following lemma.

**Lemma** **1** ([29]). *With explicit consideration of actuator faults, the faulty τ in (1) is rewritten as*

(3) $$\begin{array}{c} \tau =\tau^{★}+f_{\tau }, \end{array}$$

*where $\tau^{★}\in R^{6\times 1}$ is the required dynamic input, and $f_{\tau }$ contains unknown fault factors that can be estimated by*

(4) $$\begin{array}{c} {\hat{f}}_{\tau }=x+PM\nu , \end{array}$$

*where ${\hat{f}}_{\tau }$ is the estimation, x is a state vector that satisfies*

(5) $$\begin{array}{c} \dot{x}=-P\left(x+PM\nu -C\left(\nu \right)\nu -D\left(\nu \right)\nu -g\left(\eta \right)+\tau^{★}\right), \end{array}$$

*and $P\in R^{6\times 6}$ is a positive definite diagonal matrix.*

The varied elements in *B* are unknown, which could make the linear relationships in (2) nonlinear, where feature confusions that indicate confused correspondences between the fault features and actual fault are inevitable. Therefore, FL for the faulty actuator based on general methods is difficult to be achieved, but an early and effective FL is useful for supporting active FTC. If some relationships between $f_{\tau }$ and the faulty actuators are distinguishable beyond (2), the faulty actuator may still be located. The following section will reveal the relationships by analyzing the features of the fault parameters, including ‘+’ (positive), ‘−’ (negative), ‘0’ (zero), ‘≠’ (nonzero), and ‘×’ (uncertain).

**Remark** **1.** *Noise is not directly expressed in (4) and (5). As they could be easily transferred to ${\hat{f}}_{\tau }$ from the observed ν and η, a vector $d_{\hat{f}}$ that represents the noise and is restricted by $\left|\right|d_{\hat{f}}{\left|\right|}_{\infty }\leq d_{0}<\infty$ could be added. With $d_{\hat{f}}$, fault thresholds should be considered to use these features [30]. Since the main results are generated based on $f_{\tau }$, and $d_{\hat{f}}$ can be easily added, the following analyses will first consider $d_{0}=0$ and point out the differences later.*

**Remark** **2.** *By default, the following fault $f_{\tau }$ is obtained from Lemma 1. The additive descriptions for actuator faults could be replaced by proportional ones; however, in the current research, the former is chosen.*

## 3. Main Results

Obstacles that have relative motions and cannot be avoided can impact the AUV and produce deformations or fractures on the AUV hull or the actuators deployed outside the hull. Unpredictably, sensitive elements, control components, and inside actuators may also undergo partial or completely damaging faults caused by water infiltration or other complex phenomena. Any faults occurring on these parts are likely to bring unnecessary forces and torques into the system. The following subsections locate the faults by analyzing fault features and developing FL strategies.

Sensor faults can be isolated through redundant sensitive strategies based on existing results [31,32] and are not considered to produce feature confusion hereafter. The deformation and fracture faults on the hull can also be reasonably neglected since the hull has a streamline shape, and velocity-limited impact from the sea will not cause apparent damage compared to the one on actuators; an example can be seen in [33], where a white shark attacked the AUV and left visible bite marks, but the hull kept the AUV safe.

The subsequent methods will be produced based on the given model (1). The actuator fault usually occurs separately, since the actuators are not deployed intensively in principle and since external or internal causes rarely cause simultaneous faults. The state observation under the sea current should be implementable for the following results. Accordingly, the following assumptions are proposed.

**Assumption** **1.** *The dynamic model of the AUV is assumed to be completely known.*

**Assumption** **2.** *Faults of different actuators are assumed to occur separately; meanwhile, the faulty actuator does not fail and can still carry out some degrees of movement.*

**Assumption** **3.** *The fault parameters can always be observed, even in the presence of sea current.*

### 3.1. Fault Features of the Propulsion System

The propulsion system, mainly shown in Figure 1b, contains the propulsive motor, propeller blades, a transmission shaft, a rotation controller, and some other transmission components, in which the faults may change the propeller rotation speed and induce undesired AUV motions. The faults are considered to occur inside and outside the hull, respectively. The inside faults are under the hull’s protection, whereas the outside ones might induce disproportionate relationships between forces and torques, as structure deformation, fracture, or material addition could exist.

(1) Fault features inside the hull

As the shaft is a bridge of the motor and propeller, which is also protected by the hull, the part of the propulsion system inside the hull excludes the propeller blades. If any propulsion faults occurred inside the hull and did not damage the AUV completely, the fault only affects the shaft rotation speed and hence changes the propulsion force and torque along axis $x_{b}$. A proposition is concluded for extracting features of the propulsion fault occurring inside the hull.

**Proposition** **1.** *If any propulsion fault occurs inside the hull and changes the propeller rotation speed, the generated forces and torques are changed; however, the additive forces and torques are all 0 when the desired forces and torques from $\tau_{★}$ are designed, based on the actual rotation speed of the propeller.*

Proposition 1 is apparent because the desired forces and torques adjusted to fit the current propeller rotation speed are inevitably equal to the actual ones while the propeller outside the hull is fault-free.

**Remark** **3.** *Being limited to the uncertainty of attainable information inside the hull, further FL for the propulsion system inside the hull will not be investigated hereafter.*

(2) Fault features outside the hull

**Figure 2:**
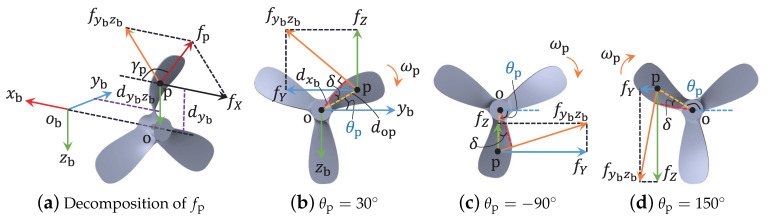
Periodically additive forces on the deformed blade, where (**a**) gives the decomposition of the additive resultant force $f_{p}$, meanwhile, (**b**–**d**) present the force decompositions at the rotation angles of $\theta_{p}=30^{\circ }$, $\theta_{p}=-90^{\circ }$, and $\theta_{p}=150^{\circ }$, respectively.

When part of the propulsion system outside the hull encounters a fault, the fault rotates with the same frequency of the propeller. As long as the fault is not axisymmetric on axis $x_{b}$ (where an axisymmetric fault is too special to be considered), time-varying and periodically additive forces and torques along axes $y_{b}$ and $z_{b}$ are produced; that is, the related elements of $f_{\tau }$ will change periodically. Figure 2 illustrates an example.

Usually, the rotation cycle of a propeller is short when an AUV is sailing in a normal velocity; the transient features of $f_{\tau }$ are difficult to be clearly captured by the AUV embedded system. However, the periodical features of $f_{\tau }$, as shown in the following proposition, are relatively stable in further research on the condition that the propeller rotation speed in each statistical cycle remains the same.

**Proposition** **2.** *If one piece of the propeller blades has become faulty, the time integrations of elements from $f_{\tau }$, except $f_{X}$ and $f_{K}$, in a rotation cycle equal 0 simultaneously under a constant rotation speed.*

**Proof.**  Without loss of generality, the blade deformation given in Figure 2 is set to describe the propeller fault that affects the AUV’s motion. At time instant $t_{p}$, the additive forces are

(6) $$\begin{array}{c} \left\{\begin{array}{c} f_{X}=f_{p}\cos\gamma_{p},\\ f_{Y}=f_{p}\sin\gamma_{p}\sin\left(\theta_{p}\left(t\right)-\delta \right),\\ f_{Z}=-f_{p}\sin\gamma_{p}\cos\left(\theta_{p}\left(t\right)-\delta \right), \end{array}\right. \end{array}$$

where $f_{p}$ is the resultant force, $\gamma_{p}$ is the angle between $f_{p}$ and axis $x_{b}$, δ is a positive included angle determined by the additive force $f_{y_{b}z_{b}}$ (defined as $f_{p}\sin\gamma_{p}$), and $\theta_{p}\left(t\right)\in \left(\pi ,\pi \right]$ represents the clockwise angle between the dashed segment op and the positive direction of $o_{b}y_{b}$ in plane $y_{b}o_{b}z_{b}$.

When the speed variation of the propeller rotation is negligible, the magnitudes of $f_{p}$ and $\gamma_{p}$ remain the same; thus, the time integration $F_{X}$ of $f_{X}$ in a rotation cycle is not zero. However, $\theta_{p}\left(t\right)$ varies from −π to π continuously and periodically. Thus, the time integrations of $f_{Y}$ and $f_{Z}$ in a rotation cycle are

(7) $$\begin{array}{c} \left\{\begin{array}{cc} F_{Y} & =\int_{t_{p}}^{t_{p}+t_{c}}f_{Y}dt=0,\\ F_{Z} & =\int_{t_{p}}^{t_{p}+t_{c}}f_{Z}dt=0, \end{array}\right. \end{array}$$

where $t_{c}\geq t_{c|\min}$ is the cycle time, and $t_{c|\min}$ indicates the minimal cycle time.

For the torques, $f_{K}$ defined along axis $x_{b}$ is

(8) $$\begin{array}{c} f_{K}=f_{y_{b}z_{b}}d_{x_{b}}, \end{array}$$

where $d_{x_{b}}$ is the distance from $f_{y_{b}z_{b}}$ to axis $x_{b}$. The positive or negative feature of $f_{K}$ can be exactly known for the faulty situation by using the right-hand screw rule.

Torque $f_{M}$ is produced by forces $f_{X}$ and $f_{Z}$ along the axis $y_{b}$. Based on Figure 1a and Figure 2, the part of $f_{M}$ generated by $f_{X}$ is

(9) $$\begin{array}{c} f_{M_{X}}=f_{X}d_{y_{b}}=-f_{X}d_{\mathrm{op}}\sin\theta_{p}\left(t\right), \end{array}$$

where $d_{y_{b}}$ is the distance from $f_{X}$ to axis $y_{b}$; the part generated by $f_{Z}$ is

(10) $$\begin{array}{c} f_{M_{Z}}=f_{Z}d_{y_{b}z_{b}}=-f_{p}d_{y_{b}z_{b}}\sin\gamma_{p}\cos\left(\theta_{p}\left(t\right)-\delta \right), \end{array}$$

where $d_{y_{b}z_{b}}$ describes the distance between $f_{Z}$ and axis $y_{b}$. Obviously, the features of $f_{M_{X}}$ and $f_{M_{Z}}$ also change with periodical $\theta_{p}\left(t\right)$; thus, the time integration of $f_{M}$ in a rotation cycle is

(11) $$\begin{array}{c} F_{M}=\int_{t_{p}}^{t_{p}+t_{c}}\left(f_{M_{X}}+f_{M_{Z}}\right)dt=0. \end{array}$$

The feature of torque $f_{N}$ produced by forces $f_{X}$ and $f_{Y}$ along the axis $z_{b}$ is similar to $f_{M}$, and the time integration in a rotation cycle is also 0. The proof is thus completed. □

Propositions 1 and 2 provide necessary features when faults occur in the propulsion system. The sufficiency will be supplied when the mutually exclusive features are given based on the following fault analyses.

**Remark** **4.** *For any deformed faults of actuators, an acoustic sensor might be helpful. However, it is difficult to deploy enough acoustic sensors for all actuators.*

### 3.2. Fault Features of the Attitude Control System

The attitude control system (specifically referred to as the rudder system in this paper, as shown in Figure 1b) consists of a steering engine, rudder surfaces, rudder stocks, an attitude controller, and some other transmission components. The lift force produced by each rudder surface against the coming water flow supports the AUV attitude control; therefore, any fault that can change the forces or torques will induce an undesired attitude. The faulty rudder might swing among a small scope of angles where the frequency cannot be as high as the propeller or stuck at a fixed place with relatively stationary properties. The features of $f_{\tau }$ are apparently different from the propulsion system, and analyses are carried out separately for faults inside and outside the hull for the same reason.

(1) Fault features inside the hull

The electronic or mechanical damages occurring on part of the rudder system inside the hull might affect the rotation speed and angle of the rudder surface outside the hull. However, under the hull’s protection, the dynamic relationships between the rudder surface and the water flow remain the same.

The rotations of horizontal $R_{L}$ and $R_{R}$ around respective stocks are considered to be actuated simultaneously by the same engine for controlling the AUV’s pitch attitude. Any fault occurring on related parts inside the hull lead both rudder surfaces to the same faulty angle. Therefore, the drag and lift forces provided by these two surfaces do not match the required ones; that is, $f_{X}\neq 0$ and $f_{Z}<0$ (or $f_{Z}>0$), where the additive rudder angle increases (or correspondingly decreases) the pitch angle. Meanwhile, $f_{X}$ is produced symmetrically with axis $x_{b}$ in plane $x_{b}o_{b}z_{b}$ by $R_{L}$ and $R_{R}$ and does not produce $f_{M}$ or $f_{N}$. Since no additive force is produced along axis $y_{b}$—$f_{Y}=0$ and $f_{N}=0$. Moreover, $f_{M}$ is produced by a nonzero $f_{Z}$; hence, the corresponding feature should be the same as $f_{Z}$.

The symmetrical rudders $R_{U}$ and $R_{D}$ are actuated collaboratively to control yaw and roll attitudes. Being similar to the horizontal rudders, the fault features could be obtained by analyzing the force and torque relationships. However, the fault of $R_{U}$ or $R_{D}$ inside the hull will only influence the corresponding rudder angle; hence, vertical and differential rudder angles are changed simultaneously and produce different kinds of additive forces and torques by comparing them with the horizontal faults. The following proposition is given to synthesize these fault features.

**Proposition** **3.** *If some rudder fault occurs inside the hull, exclusive fault features appear, as shown in Table 1.*

**Remark** **5.** *Rudder faults inside the hull will not produce variations to the hydrodynamic coefficients in B of Equation (2). Thus, the faulty rudder and additive rudder angle cannot be directly obtained provided that the sufficiency of these features in Table 1 is confirmed.*

(2) Fault features outside the hull

**Figure 3:**
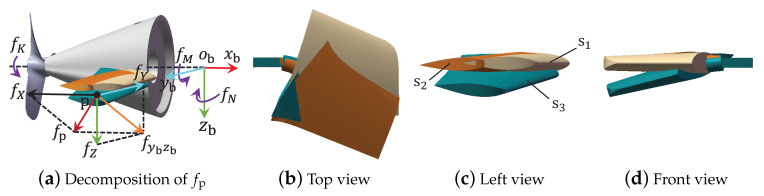
Deformed faults on $R_{R}$ outside the hull, where (**a**) reveals the decomposition of the additive resultant force $f_{p}$, (**b**–**d**) provide respectively the top, left, and front views of the rudders for comparisons, $s_{2}$ and $s_{3}$ represent respectively the deformed rudders from impacts with $0^{\circ }$ and $15^{\circ }$ initial rudder angles, and $s_{1}$ indicates a fault-free rudder.

The rudder faults of deformation, fracture, loss, and the addition of extra materials that occur outside the hull will probably change the required forces and torques. This section also concentrates on the fault features of $f_{\tau }$ rather than the specific forms of faulty rudders, since the common features of all influential faults are contained in $f_{\tau }$. The influential faults outside the hull may directly change the hydrodynamic coefficients, so the exact faults cannot be directly obtained by solving (2). To conduct the analysis, deformation is selected as an example. Figure 3 illustrates two deforming cases from finite element simulations, where vales from $s_{1}$ to $s_{3}$ indicate no fault, a deformation with $0^{\circ }$, and a deformation with $15^{\circ }$ initial rudder angles, respectively. With different initial rudder angles, the deformations are variant in countless ways (e.g., the differences between $s_{2}$ and $s_{3}$ include the deformations on the rudder surface and the rudder stock).

If the surface of the faulty rudder still coincides with the plane of an initial $0^{\circ }$ rudder angle (planes $x_{b}o_{b}y_{b}$ or $x_{b}o_{b}z_{b}$), the deformation will not create any lift force on this rudder. However, an unknown force will be produced along the direction of the stock axis, since the changed rudder aspect ratio breaks the force balance of these two symmetrical rudders along the stock axis. By analyzing the acting forces and torques from Figure 3, the fault features shown in Table 2 could be directly obtained.

Except the aforementioned cases where the rudder angles are $0^{\circ }$, the aspect ratio and rudder angle of the faulty rudder can be changed simultaneously; thus, the magnitude and orientation of the generated lift force will vary, such as the case of $15^{\circ }$ initial rudder angle in Figure 3. Force analysis provides specific fault features where the additive force $f_{Y}$ being parallel to the axis of the rudder stock points towards the AUV hull. Some other forms of deformation faults with a $15^{\circ }$ initial rudder angle can also occur where $f_{Y}$ may point outwards. Obviously, fault features for the remaining deformation faults are difficult to distinguish based on the estimates; nevertheless, $f_{X}\neq 0$, $f_{Y}\neq 0$, and $f_{Z}\neq 0$ could be confirmed against these cases. The features of $f_{K}$, $f_{M}$, and $f_{N}$ are uncertain since the accurate action point of the faulty force is unknown.

Focusing on the faults of fracture, loss, and material addition, unbalanced forces and torques with the form of $f_{\tau }$ emerge in τ. Besides the features proposed in Table 2, the others are also analogous to the deformations where $f_{X}\neq 0$, $f_{Y}\neq 0$, and $f_{Z}\neq 0$, since the rudder surface is designed irregularly and the lost or added parts on horizontal (vertical) rudders are rarely symmetrical from plane $x_{b}o_{b}z_{b}$ (correspondingly, $x_{b}o_{b}y_{b}$).

**Remark** **6.** *Actuators can take countless forms of faults, which cannot be analyzed completely. However, the faults can be assorted to the given groups based on the fault features; and FTC schemes could be designed against specific features and actuators.*

### 3.3. Active Fault Localization

The fault features of the propulsion and rudder systems are given in different forms. To compare the features in the same dimension, the time integrations for features of rudder faults are produced in the propeller rotation cycle. Table 3 collects the integrated fault features of the propulsion and rudder systems on the condition that the rotation speed and the rudder angles in each short statistical cycle remain unchanged.

**Figure 4:**
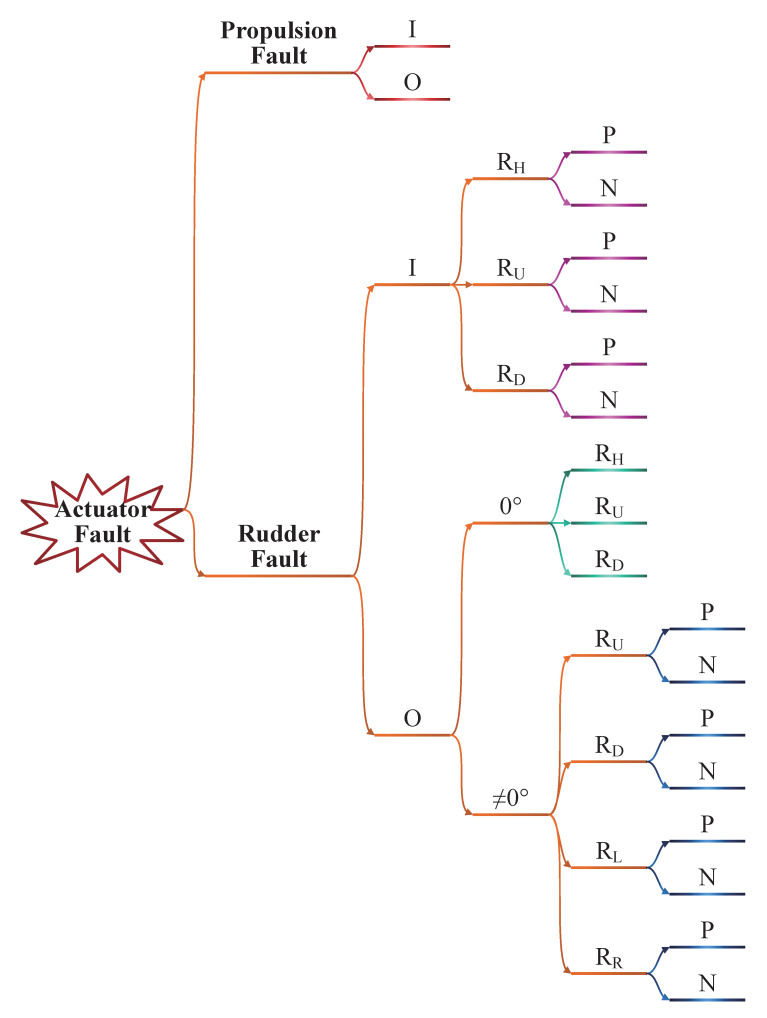
Actuator fault tree of a torpedo-shaped AUV.

Based on Table 3, Figure 4 is developed focusing on different faulty regions. Possible faulty cases that can change the motion of a given AUV are divided into 19 exclusive categories qualitatively, even though similar fault features exist as given in Table 3. Each category, described by 6 features, is a common set of countless faulty situations and can be easily distinguished from each other based on fault locations and additive directions.

The related cases from the same actuator occupy all its possible faulty directions. For instance, the cases on $R_{U}$ are divided into categories of I (inside) and O (outside), where I contains categories of P (positive direction) and N (negative direction), and O contains categories of $\alpha =0^{\circ }$ and $\alpha \neq 0^{\circ }$ (α≜ rudder angle); moreover, the category of $\alpha \neq 0^{\circ }$ is divided into P and N. Apparently, all possible fault cases are included in Figure 4, except for the ignored hull fault. Thus, the sufficiency of the fault features corresponding to the faulty regions does exist; if strategies could be established to distinguish these cases based on limited fault features, the fault could be confirmed. A proposition is given below to primarily locate the faults.

**Proposition** **4.** *With the integral features listed in Table 3, if the propeller rotation speed has been changed and the fault features are all 0 against the current rotation speed, the part of the propulsion system inside the hull became faulty; otherwise,*

(1) *If $F_{X}\neq 0$ and $F_{Y}=F_{Z}=0$, the part of the propulsion system outside the hull is faulty.*
(2) *If $F_{Y}=0$ and $F_{Z}\neq 0$, the fault occurred on the part of the horizontal rudder inside the hull or the part of the vertical rudder outside the hull.*
(3) *If $F_{Y}\neq 0$, $F_{Z}=0$, and $F_{K}\neq 0$, the part of the vertical rudder corresponding to the specific features of $F_{Y}$ and $F_{K}$ inside the hull is faulty.*
(4) *If $F_{Y}\neq 0$, $F_{Z}=0$, and $F_{K}=0$, the horizontal rudder outside the hull is faulty.*
(5) *If $F_{Y}\neq 0$ and $F_{Z}\neq 0$, the part of some rudder outside the hull is faulty and could not be localized any further, based on Table 3.*

Since the strategies in Proposition 4 were directly extracted from Table 3, the proof is omitted. Items (1), (3), and (4) possess exclusively corresponding relationships between the fault features and the faulty regions. Item (2) can be further resolved based on the following active FL strategy.

**Proposition** **5.** *Given $F_{Y}=0$ and $F_{Z}\neq 0$, the horizontal rudder angle with any acceptable nonzero lift force can be changed by using (2). If the newly acquired $f_{Z}^{†}\neq f_{Z}$, the faulty actuator is the part of the horizontal rudder inside the hull. Otherwise, the vertical rudder that has a $0^{\circ }$ rudder angle outside the hull can be changed to any other angle. If the newly acquired $f_{Y}^{†}=0$, the faulty actuator is still the part of the horizontal one inside the hull; otherwise, the former vertical rudder outside the hull is faulty.*

**Proof.**  If the fault is on the vertical rudder, the control strategy on the horizontal rudders will not change the rudder angle or the fault degree of the faulty vertical rudder; thus, the additive fault $f_{\tau }$ will not change; that is, $f_{Z}^{†}=f_{Z}$, provided that the AUV sailing velocity remains the same.

When the fault is on the horizontal rudder inside the hull, the actual lift force can have forms of varying and fixed gaps from the required one. If the gap is varying, $f_{Z}^{†}\neq f_{Z}$, and the first conclusion is proved. If the gap is fixed, $f_{Z}^{†}=f_{Z}$, which means the situation is still ambiguous.

Before taking the second active step, the doubtful vertical rudder should be confirmed. Typically, there is always an angle difference between $R_{U}$ and $R_{D}$ to compensate the torque along axis $x_{b}$ produced by the propeller. Thus, only one vertical rudder can take a $0^{\circ }$ rudder angle.

By taking the second active step, the doubtful vertical rudder with a nonzero rudder angle will definitely produce $f_{Y}^{†}\neq 0$ if it has a fault, because the deformed vertical rudder cannot generate the required force precisely. In other words, $f_{Y}^{†}=0$ indicates that the horizontal rudder inside the hull is faulty. The proof is thus completed. □

When fault features in Item (5) of Proposition 4 are acquired, an active FL strategy given in the following proposition can identify the actual faulty rudder outside the hull.

**Proposition** **6.** *If the fault has been restricted to the part of the rudder outside the AUV hull with a nonzero rudder angle, any control inputs to eliminate part of $f_{Y}$ (or $f_{Z}$) by adjusting vertical (or, correspondingly, horizontal) rudders can be implemented. Given the newly acquired $f_{Y}^{†}\neq f_{Y}$ (or $f_{Z}^{†}\neq f_{Z}$), the vertical (or horizontal) rudders are faulty; otherwise, the horizontal (or vertical) rudders are faulty.*

Proposition 6 stands because the operation on a fault-free rudder will not induce any additive fault, whereas any operations on a faulty rudder can simultaneously cause the fault to change. Faults with a fixed gap to vary on the faulty rudder outside the hull cannot always hold because the designed dynamic relationships are changed. When any faults on the vertical rudders are confirmed, further localization against $R_{U}$ and $R_{D}$ can be realized based on the following strategy.

**Proposition** **7.** *When the fault has been confirmed on the vertical rudders outside the hull, the vertical rudder can be chosen; it can produce a relatively large but opposite force against $f_{Y}$ along axis $y_{b}$ to turn an appropriate angle to provide additive $-f_{Y}$. If the newly acquired $f_{Y}^{†}\neq f_{Y}$, the adjusted vertical rudder is faulty; otherwise, the opposite one is faulty.*

Proposition 7 is analogous to Proposition 6. If the fault on horizontal rudders has been confirmed, it is not necessary to identify the fault between $R_{L}$ and $R_{R}$ since they are controlled by one engine, and the localization is not very useful for the active FTC.

**Remark** **7.** *The current research assumes that both the left and the right horizontal rudder surfaces are controlled by the same engine. When these surfaces are driven separately, the findings can be directly improved based on the aforementioned analyzing processes.*

**Remark** **8.** *The implementations of Propositions 5 to 7 that involve rotary movements of the faulty rudder may bring unstable factors for that the active control does not belong to FTC, whereas the costs make an early FL possible to support effective FTC for stopping the losses in time. The rotary movements of the fault-free rudders will only affect the AUV attitudes without affecting the system stability.*

### 3.4. Generality

Noise is difficult to avoid in view of the present technical level. Since the aforementioned methods were developed to compare fault parameters or their integration with 0, relevant relationships in the aforementioned methods can be improved with bounded noise ($d_{0}\neq 0$). A corollary can be generated as follows.

**Corollary** **1.** *With bounded noise $|d_{i}|\leq d_{i|\max}$(i=1,⋯,6) corresponding to $f_{i}\in f_{\tau }$ from the proposed methods,*

(1) *the relationships ‘+’, ‘−’, ‘=’, and ‘≠’ between any $f_{i}$ and 0 should be changed to ‘$f_{i}\in \left(d_{i|\max},+\infty \right)$’, ‘$f_{i}\in \left(-\infty ,-d_{i|\max}\right)$’, ‘$f_{i}\in \left[-d_{i|\max},d_{i|\max}\right]$’, and ‘$f_{i}\notin \left[-d_{i|\max},d_{i|\max}\right]$’, respectively; regarding the integration of $f_{i}$, $d_{i|\max}$ should be replaced by $D_{i|\max}≜\int_{t_{p}}^{t_{p}+t_{c}}$$d_{i|\max}dt$;*
(2) *the relationships ‘=’ and ‘≠’ between any $f_{i}$ and $f_{j}$(j=1,⋯,6;j≠i) should be changed to ‘$f_{i}-f_{j}\in \left[-2d_{i|\max},2d_{i|\max}\right]$’ and ‘$f_{i}-f_{j}\notin \left[-2d_{i|\max},2d_{i|\max}\right]$’, respectively.*

**Remark** **9.** *The feasibility of (1) in Corollary 1 is obvious as the noise boundary takes the place of 0. Toward (2) in Corollary 1, a multiple of 2 is given, since $f_{i}$ and $f_{j}$ both have noise from $\left[-d_{i|\max},d_{i|\max}\right]$ and the subtraction produces $\left[-2d_{i|\max},2d_{i|\max}\right]$ directly. Based on Corollary 1, the proposed methods are robust to bounded noise.*

The FL methods for typical robots with redundant thrusters have been given in [29], whereas previously proposed methods in this paper only correspond to the typical torpedo-shaped AUVs that have four rudders and one thruster. Without a loss of generality, a constructive scheme is proposed.

**Proposition** **8.** *To actively locate the faulty actuator from a robot with simultaneous control surfaces and thrusters, a series of measures are adoptable:*

(1) *construct the dynamic model (1) and extract the fault parameters given in (3);*
(2) *produce a table of integrated fault features (Table 3) and an actuator fault tree (Figure 4) to promise sufficiency and necessity based on structure and motion analyses;*
(3) *establish an FL strategy to primarily locate faults based on the table and the fault tree;*
(4) *give specialized control inputs to one of the feature-confused actuators to locate the actual faulty one.*

**Remark** **10.** *Simultaneous faults, which have not been considered since Assumption 2, can be covered by extending previous methods based on their thoroughly discovered fault features.*

**Remark** **11.** *The time consumption of FL can be counted as $t_{c}+t_{\mathrm{pl}}+t_{\mathrm{al}}$, where $t_{c}$ is given in (7), $t_{\mathrm{pl}}$ ($t_{\mathrm{pl}}\geq t_{\mathrm{pl}|\min}$) is the execution time for primary FL from Proposition 4, with $t_{\mathrm{pl}|\min}$ being the minimal time of comparison calculation, and $t_{\mathrm{al}}$ ($t_{\mathrm{al}}\geq t_{\mathrm{ctrl}|\min}+t_{\mathrm{est}|\min}+t_{\mathrm{cal}|\min}$) is the execution time for active FL based on Propositions 5–7 with $t_{\mathrm{ctrl}|\min}$, $t_{\mathrm{est}|\min}$, and $t_{\mathrm{cal}|\min}$ being, respectively, the minimal time consumptions for control execution, fault estimation, and comparison calculation.*

## 4. Simulation

Simulations are given for illustrating the effectiveness of the proposed methods, including the features of propeller faults outside the hull and Proposition 6. Since the fault features for other actuator faults are apparent, and Propositions 5–7 are given based on analogous principles, illustrations of the effectiveness of the remaining methods are omitted. The following simulations were taken based on finite element analysis in the software environment ANSYS Workbench R19.0, where the deformation was generated by the Workbench LS-DYNA, and the fluid forces were produced by the Fluent. The mesh for the AUV in Workbench LS-DYNA contains nodes 26108 and elements 106949, whereas the nodes and elements of the mesh in Fluent are 106779 and 568103, since the mesh of a fluid cylinder for the AUV to sail was taken into account.

### 4.1. Propulsion Fault Features Outside the Hull

**Figure 5:**
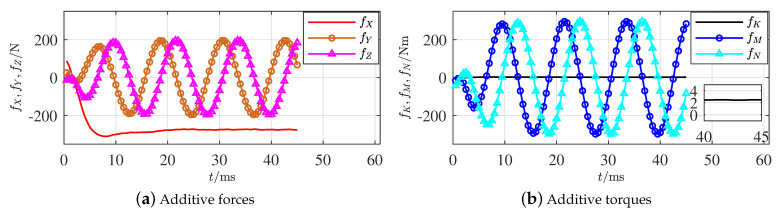
Additive forces and torques of propeller faults outside the hull.

A propeller deforming fault given in Figure 2 was made after the torpedo-shaped AUV shown in Figure 1a impacted a solid barrier under a sailing speed of 5 m/s and a propeller rotation speed of 5000 rpm. A subsequent hydrodynamic calculation in a short moment was carried out with the same sailing and rotation speeds; accordingly, the additive forces and torques from the deformed blade were recorded, as shown in Figure 5a,b, respectively.

For the propeller fault outside the hull, only $F_{X}$ of the 6 integration features in a rotation cycle is definitely nonzero based on the generated result in Table 3. Apparently, based on Figure 5a, $f_{X}<0$, which indicates that the integration will not be 0 and coincides with the given feature. Although the feature of $f_{K}$ is slightly greater than 0, it is far smaller than the other features and cannot be definitively affirmed. The other fault parameters varying as sine curves with cycle time 12 ms directly prove that their integration values in each rotation cycle equal 0 and coincide with the given results. The fault features of the propeller outside the hull have thus been effectively illustrated.

**Remark** **12.** *Because the simulation results were produced by ANSYS Fluent, there is still a period of time for the calculation to converge to a relatively small error; that is, the initial parts of the figures might not be accurate.*

### 4.2. Effectiveness of Proposition 6

**Figure 6:**
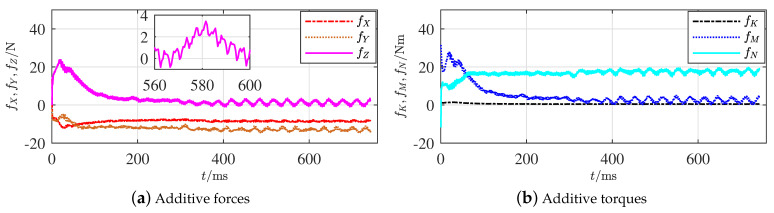
Additive forces and torques of rudder faults outside the hull.

The fault was set to be the deformation $s_{3}$ on $R_{R}$ from an impact, as shown in Figure 3, where the relative impacting speed was 5 m/s, and the propeller maintains the same rotation rate after the impact. The additive forces and torques, respectively, were recorded and are plotted in Figure 6a,b.

Around time instant 400 ms, the simulation calculation clearly converged. Apparently, the subsequent additive forces and torques remained relatively stable values. The features in Figure 6a coincide with the records from Table 3, where $F_{Y}\neq 0$ and $F_{Z}\neq 0$ are integrated, but they could not locate the actual fault. However, it was confirmed that the fault was on one part of the four rudders outside the hull. The absolute values of $f_{Z}$ are relatively smaller than others, which might be 0 in some deformation situations and coincide with the situation of Item (4) in Proposition 4.

**Figure 7:**
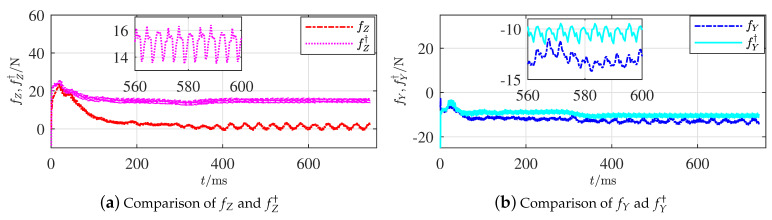
Comparisons of additive forces along axes $z_{b}$ and $y_{b}$.

Two active FL strategies from Proposition 6 were executed by adjusting the $5^{\circ }$ of both the horizontal and vertical rudders, respectively. Figure 7a,b display the comparison results of $f_{Z}$ and $f_{Y}$ from the adjusted horizontal and vertical rudders, respectively.

The calculation converged around 400 ms. Apparently, the gap between $f_{Z}$ and $f_{Z}^{†}$ is relatively larger than the one between $f_{Y}$ and $f_{Y}^{†}$. The gap between $f_{Z}$ and $f_{Z}^{†}$ definitively shows that $f_{Z}$ varied when the horizontal rudder angle was adjusted; however, the gap between $f_{Y}$ and $f_{Y}^{†}$ was relatively 0 when the angles of vertical rudders were changed. The variations of the fault features coincide with the situations given in Proposition 6 when the fault occurred on the horizontal rudders. Since the horizontal and vertical rudders make a symmetrical cross, the simulation on a faulty vertical rudder will have similar results and is omitted.

The gap between $f_{Y}$ and $f_{Y}^{†}$ does not reach 0 completely because the grid accuracy was limited in each simulation. Nevertheless, the magnitudes of these two gaps are apparently different. The effectiveness of Proposition 6 is thus illustrated. The effectiveness of the other findings in Proposition 4 is relatively evident, so the illustration simulations are omitted.

### 4.3. Generality Illustration

As only the finite element analysis was considered where data were obtained from the software directly, the influences from the measurement noise and parameter uncertainties were absent in the former simulations; moreover, the ocean current was not considered either. Comparatively, the situation described in Section 4.2 is reconsidered, whereas a current of constant velocity (1 m/s) and direction ($-z_{b}$) is added in the simulation. Meanwhile, an identical boundary ($d_{\max}=2$ N or Nm) of measurement noises for each element of $f_{\tau }$ and three additive uncertainties (10%, −10%, and 10%) on the moments of inertia towards respectively the axes $x_{b}$, $y_{b}$, and $z_{b}$, are simultaneously taken into account with the model-based parameter observation.

**Figure 8:**
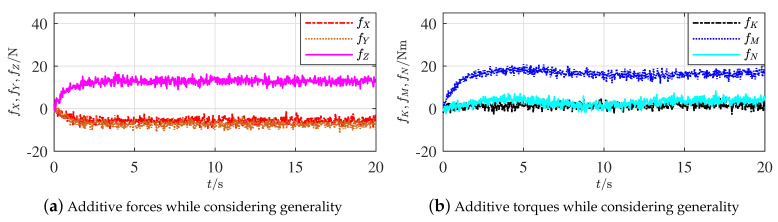
Additive forces and torques of rudder faults outside the hull while considering generality.

Figure 8a,b represent, respectively, the additive forces and torques under the given current, noise, and uncertainty. The primary segments of each curve began from 0 and the satisfactory convergence took nearly 3 s. These processes were produced by the observers and the convergent speed was a result of considering the influences of uncertainties. Apparent influences of uncertainties on the acquired data could be seen from $f_{M}$ and $f_{N}$, whereas $f_{M}$ and $f_{N}$ are not important in the proposed methods of this paper. The influences of measurement noises are also present in these curves, which have not confused the data yet under the given boundary.

**Figure 9:**
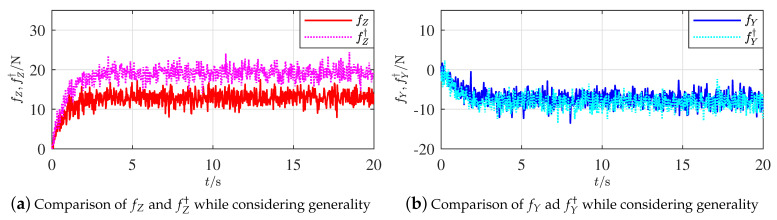
Comparisons of additive forces along axes $z_{b}$ and $y_{b}$ while considering generality.

By following Proposition 6, Figure 9a,b show, respectively, comparisons of $f_{Z}$ and $f_{Y}$ from the correspondingly adjusted horizontal and vertical rudders. Being similar to Figure 7a,b, the effectiveness of Proposition 6 is thus illustrated, when current, measurement noise and parameter uncertainty are simultaneously included.

## 5. Conclusions

This paper focused on localizing actuator faults on torpedo-shaped AUVs. Analyses were carried out based on the known nonlinear dynamic model and the actuator configuration relationships. Estimable additive parameters were set up to represent actuator faults. Fault features described by the positive or negative features of fault parameters were separately analyzed against different faulty parts of the actuators and organized in various propositions and tables. A synthesized table and a fault tree were concluded; they support the sufficiency of the given fault features and resulted in a primary and three active FL strategies that can preliminarily determine and actively locate fault regions, respectively. The generality of the proposed methods was analyzed considering measurement noise and typical robots with simultaneous control surfaces and thrusters. Simulations with propeller and rudder deformations were executed to illustrate the effectiveness of the proposed methods.

The proposed methods have thus creatively solved FL issues where fault features can be confused. Some work is still left for intensive research in the future, such as FTC design and real experiments. For the latter, an AUV that works for marine ranching and verifies the proposed methods is being produced using funds related to this research. 

## Figures and Tables

**Table 1:** Features of the faults from the rudder system inside the hull.

Rudder	Direction	$f_{X}$	$b_{Y}$	$f_{Z}$	$f_{K}$	$f_{M}$	$f_{N}$
$R_{H}$	P	≠	0	−	0	−	0
	N	≠	0	+	0	+	0
$R_{U}$	P	≠	−	0	−	≠	+
	N	≠	+	0	+	≠	−
$R_{D}$	P	≠	−	0	+	≠	+
	N	≠	+	0	−	≠	−

R_H_: combination of R_L_ and R_R_; P (positive) and N (negative): equivalent directions of rudder angles based on additive faults.

**Table 2:** Fault features of the rudder system outside the hull with $0^{\circ }$ rudder angles.

Rudder	$f_{X}$	$f_{Y}$	$f_{Z}$	$f_{K}$	$f_{M}$	$f_{N}$
$R_{H}$	≠	≠	0	0	0	≠
$R_{U}$	≠	0	≠	0	≠	0
$R_{D}$	≠	0	≠	0	≠	0

**Table 3:** Integrated fault features of propeller and rudders.

Actuator	Region	Direction	$F_{X}$	$F_{Y}$	$F_{Z}$	$F_{K}$	$F_{M}$	$F_{N}$
$P_{S}$	I	—	0	0	0	0	0	0
	O	—	≠	0	0	×	0	0
$R_{H}$	I	P	≠	0	−	0	−	0
	I	N	≠	0	+	0	+	0
	O	$0^{\circ }$	≠	≠	0	0	0	≠
$R_{L}$	O	P	≠	≠	≠	×	×	×
	O	N	≠	≠	≠	×	×	×
$R_{R}$	O	P	≠	≠	≠	×	×	×
	O	N	≠	≠	≠	×	×	×
$R_{U}$	I	P	≠	−	0	−	≠	+
	I	N	≠	+	0	+	≠	−
	O	$0^{\circ }$	≠	0	≠	0	≠	0
	O	P	≠	≠	≠	×	×	×
	O	N	≠	≠	≠	×	×	×
$R_{D}$	I	P	≠	−	0	+	≠	+
	I	N	≠	+	0	−	≠	−
	O	$0^{\circ }$	≠	0	≠	0	≠	0
	O	P	≠	≠	≠	×	×	×
	O	N	≠	≠	≠	×	×	×

P_S_: propulsion system; I and O: faults inside and outside the hull.

## Abbreviations

The following abbreviations are used in this manuscript:

AUV	autonomous underwater vehicle
FTC	fault-tolerant control
FD	fault diagnosis
FL	fault localization
